# A concealed inguinal presentation of a gastrointestinal stromal tumor (GIST): a case report and literature review

**DOI:** 10.1186/s12893-021-01088-4

**Published:** 2021-03-03

**Authors:** Yujie Yuan, Li Ding, Min Tan, An-jia Han, Xinhua Zhang

**Affiliations:** 1 Center of Gastrointestinal Surgery, The First Affiliated Hospital of Sun Yat-Sen University, 58 2nd Zhongshan Road, Guangzhou, 510080 Guangdong People’s Republic of China; 2 Department of Pathology, The First Affiliated Hospital of Sun Yat-Sen University, 58 2nd Zhongshan Road, Guangzhou, 510080 Guangdong People’s Republic of China

**Keywords:** Gastrointestinal stromal tumor, Groin hernia, Surgery, Immunostains, Literature review

## Abstract

**Background:**

Gastrointestinal stromal tumor (GIST) can arise anyplace along the gastrointestinal (GI) tract. The uncommon tumor location in groin area is rarely reported.

**Case presentation:**

We herein reported a metastasized case presented as GI hemorrhage complicated with indirect hernia, and underwent tumor cytoreduction, herniorrhaphy and chemotherapy for jejunal GIST. The case was described consecutively based on the process of surgical management, with a good follow-up result. A literature review by searching similar case reports from two national medical databases was performed to summarize clinical features of such unusual presentation of GIST, which included hernia characteristics, short- and long-term outcomes of this disease. It showed GIST presenting as groin hernia was rarely reported and all available 11 cases suggested a primary tumor and required both tumor resection and hernia repair. The long-term results indicated 64.3% overall survival at 5 years after the incidental diagnosis.

**Conclusions:**

Inguinal hernia is an extremely rare presentation of GIST, with limited case reports available in the literature. A radical involving tumor resection plus hernia repair is an optimal surgical approach for such uncommon condition. An adjuvant medication mounting on mutated KIT gene should be strictly followed for high risk cases.

## Background

Gastrointestinal stromal tumor (GIST) is one of the most common mesenchymal tumors, and it involves the whole gastrointestinal (GI) tract. The recent advances in tissue evaluation and molecular analysis have earned a deeper understanding of GIST lesion, which was considered as a disease arising from neurogenic or smooth muscle [1]. Histologically, GIST is resulted from an anomaly transformation in the interstitial Cajal cells, responsible for motor function of the GI tract [2]. Around 80% of GISTs harbor KIT gene mutations, which would result in abnormally activation of the KIT receptor that further leads to tumor growth. Tyrosine kinase inhibitors, such as imatinib and sunitinib, are specifically effective to limit its growth and fit for unresectable, metastatic or recurrent GIST [3, 4]. Up to the present, a radical resection of whole tumor with at least 2 mm safe margin remains the golden standard to cure this disease. However, at least 50% of patients develop recurrence or metastasis [5].

The most common primary locations of GISTs are the stomach (60%) and small intestine (35%). Therefore, the dominant symptoms include upper abdominal pain, GI bleeding and passage disorders. Such non-specific features could identify 25% of GIST patients through an incident diagnosis. Imaging modalities including endoscopic ultrasound (EUS) and computed tomography (CT) play a critical role in its clinical diagnosis. However, tumor biopsy prior to surgery is not recommended, as it carries the risk of tumor rupture or seeding in the biopsy tract [6].

There is little literature on the GIST located in the hernial sac. We herein present the case of an elderly gentleman who referred to our institution for GI hemorrhage due to a ruptured GIST in small intestine with an incidental finding of a seeding tumor located in his right groin. After that, a literature review of GIST-involving inguinal hernia is performed to characterize clinical characteristics of such rare situation.

## Case presentation

**Fig. 1:**
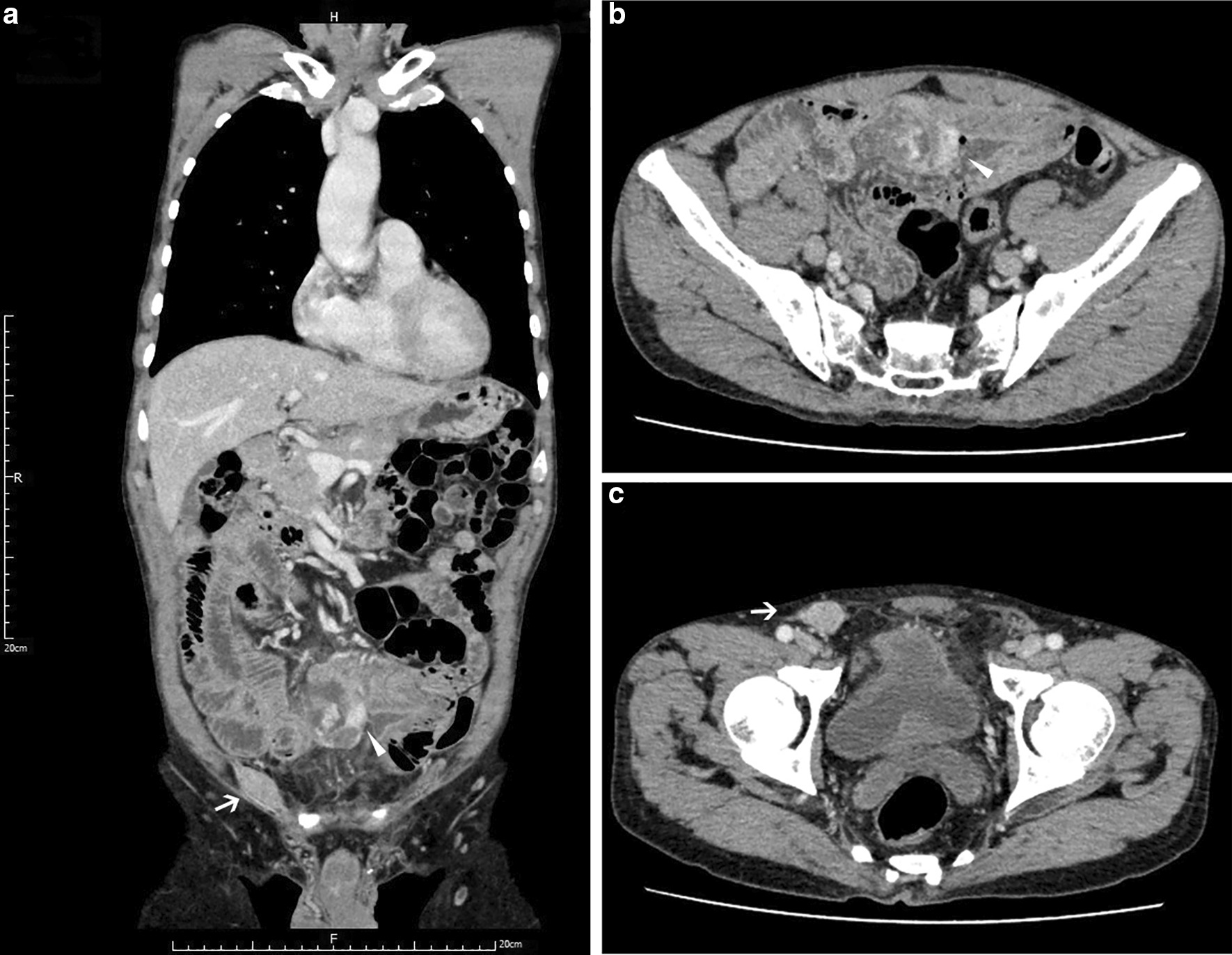
The radiological finding of the current case. CT images in the venous phase indicating a huge mass in the small intestine and an isolated inguinal mass in the right groin. Both masses were contrast-enhanced, without distinct lymph node metastasis in the abdomen. **a** Coronal plane rebuilt scan; **b**, **c** Horizontal plane scans; Arrows and triangle shapes indicating inguinal and intra-abdominal mass, respectively

A 71-year-old man presented to our emergency center with a history of diffused abdominal pain with intermittent hematochezia for 38 h. The patient denied other GI or genitourinary complaints, had an unremarkable medical history, and did not undergo prior abdominal surgery. On physical examination, the abdomen appeared tender with extensive rebound tenderness. Unfortunately, his groin areas were ignored to check due to lack of specific complaints. The blood investigation revealed a decreased hemoglobin level (9.6 g/dL). A contrast-enhanced CT scan indicated a lower GI bleeding resulting from a ruptured mass (5.2 × 4.1 × 3.0 cm^3^) located in small intestine. Meanwhile, CT imaging showed a minimal mass (2.0 × 1.5 × 0.5 cm^3^) in his right groin but failed to mention in the emergent report (Fig. 1).

The patient was immediately managed with an emergent laparotomy in light of the hemodynamic instability. During the surgery, the primary tumor was found arising from the third jejunal segment (220 cm distant from the duodenal-jejunal flexure), with disseminated, multi-focal progression of tumor seeding recorded. A palliative resection of primary tumor and seeding tumors (> 2 mm) in abdomen was achieved (R1 resection), followed by a side-to-side bowel anastomosis. The patient was discharged at the 11th post-operative day without any complications. The histopathologic report of primary tumor suggested a GIST of spindle cell nature, with high mitotic count (25/50 HPF) and significant tumor necrosis (Additional file 1: Fig. S1). The immunohistochemical (IHC) staining evaluation indicated severe positivity for CD117, DOG-1 and SDHB, mild positivity for actin and desmin, but negative for CD34 and S-100. The Ki-67 index was 20%. The molecular analysis reported a mutation rate of 18.22% for the c-KIT, mainly located on A502_T503dup *exon 9*. The tumor was hence categorized as high-risk jejunal GIST.

**Fig. 2:**
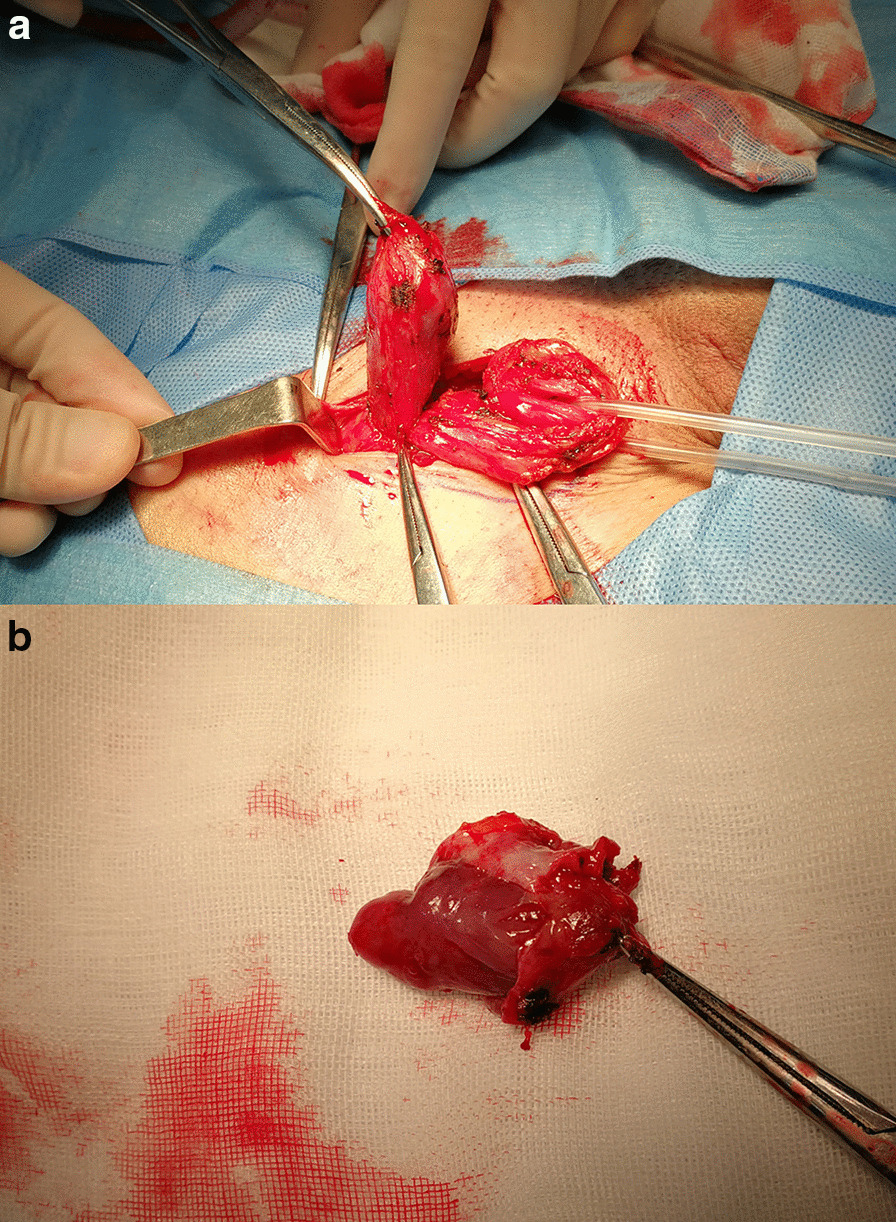
The definitive operation for his right inguinal mass. Lichtenstein approach applied to excise the mass and to repair with a self-gripping mesh. The mass along with hernia sac (**a**) was removed, with spermatic cord reserved. The tumor (**b**) was homogeneous firm as the primary GIST in the jejunum

**Fig. 3:**
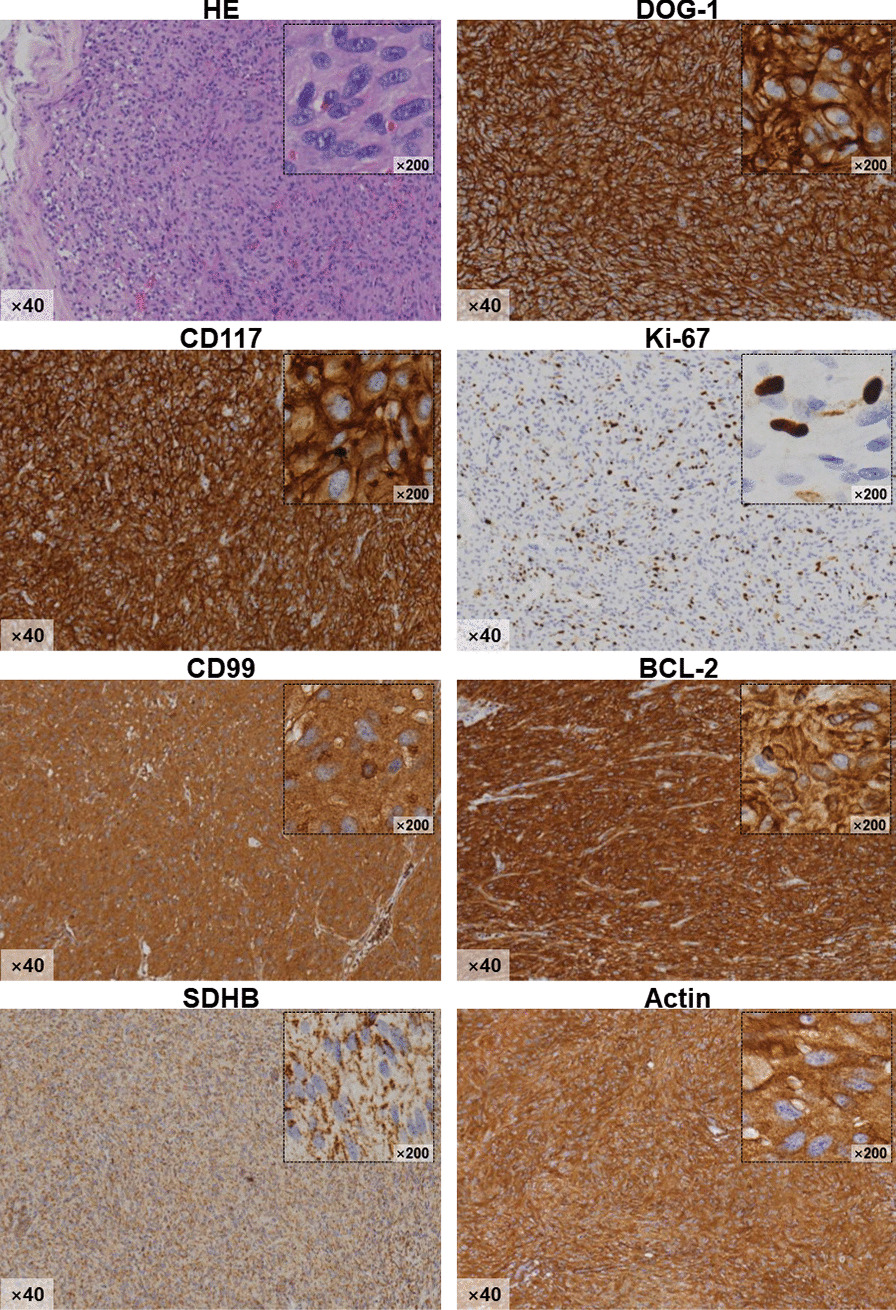
The pathological features of resected mass in the right groin. Specific pathological staining methods (HE and IHC) were used to confirm the diagnosis of GIST metastasized to the right groin

The patient received imatinib chemotherapy (400 mg/day) postoperatively for three months until a complaint of persistent pain in the right groin. On physical examination, a painful, irreducible, non-pulsatile mass (2.0 × 2.0 × 1.0 cm^3^) was felt in right groin, with negative finding in contralateral side. He was referred to the hernia unit of our department. Additional CT imaging was performed to exclude abdominal recurrence. Afterward, a definitive herniorrhaphy with Lichtenstein’s approach was accomplished (Fig. 2), with the mass along with sac removed before placing a self-gripping polyester mesh (TEM1208GR, Parietex ProGrip™, US). The intraoperative diagnosis was right indirect inguinal hernia, Gilbert II classification [7]. The patient was discharged at the second post-operative day. The final pathological report of resected specimen in his groin indicated a metastasized GIST in inguinal hernia. The tumor was spindle cells morphology, with high mitotic count (> 40/50 HPF) and strongly positive immunostains of CD117, DOG-1, Bcl-2 and CD99. The Ki-67 index was 30%, with partial positivity for SDHB (Fig. 3).

The patient continued the imatinib chemotherapy as mentioned ahead, with planed outpatient clinic visits scheduled. At the last follow-up visit on February 14th, 2020, he was survived and capable of daily work, without a recurrent inguinal hernia observed.

## Discussion

To review this unusual presentation, we performed a literature research in two national databases (PubMed and CNKI) with following mesh terms: “gastrointestinal stromal tumor”, “GIST”, “inguinal hernia”, “groin hernia”, and “case report”. The returned results were reviewed by two surgeons (Y.Y. and Z.X.) independently, with included cases summarized in Table 1. Follow up data were requested from corresponding authors by letters or mails when necessary.

**Table 1:** Clinical characteristics of cases of GIST-associated groin hernia

Study	Age and gender	Hernia location	Tumor size, cm	Imaging method	Primary or Secondary	Primary location	GIST treatment	Hernia repair
Yuan 2020	71 M	Right (G)	5.2	CT	Secondary	Jejunum	Palliative resection	Mesh
Ferhatoglu 2018 [12]	67 M	Left (G)	13.0	CT	Secondary	Ileum	Palliative resection and Imatinib	Mesh
Campbell 2017 [26]	53 M	Right (G)	9.5	CT	Secondary	Jejunum	Palliative resection, Imatinib, Sunitinib, Regorafenib and tumor cytoreduction	Mesh
Massani 2017 [10]	74 M	Right (G)	7.0	CT	Primary	Ileum	Radical right hemicolectomy	Mesh
Agrawala 2017 [27]	45 F	Right (G)	6.0	US	Primary	Ileum	Radical enterectomy and partial bladder resection and Imatinib	Tissue
Tinoco-Gonzalez 2015 [28]	50 M	Left (G)	6.0	XR	Primary	Jejunum	Palliative resection	Mesh
Zyluk 2015 [16]	71 M	Right (Fe)	7.0	XR	Primary	Ileum	Radical resection	Tissue
Liu 2014 [29]	82 M	Right (G)	4.0	CT	Secondary	Jejunum	Palliative resection and Imatinib	Tissue
Chen 2013 [30]	60 M	Right (G)	28.5	CT	Primary	Stomach	Radical resection Imatinib	Mesh
Mulla 2007 [25]	80 M	Right (G)	5.0	CT	Primary	Ileum	Biopsy and Imatinib	None
Goyal 2003 [31]	72 M	Right (G)	9.5	CT	Primary	Ileum	Radical enterectomy	Tissue

*M* male, *F* female, *G* groin, *Fe* femoral canal, *CT* computed tomography, *US* ultrasonography, *XR* simple X-ray

Briefly, ten reported cases together with the current case were finally included, with a mean age of 66 (range, 45–82) years. There was only one female (9.1%) patient suffering from this rare disease. Seven (63.6%) of 11 cases had a primary tumor in the groin area, whereas the rest (36.4%) had the metastatic tumors. There were ten inguinal hernias and only one femoral hernia, with three cases (36.4%) developing bowel obstruction. Most of reported cases underwent a definitive hernia repair, with only one case (9.1%) receiving biopsy alone. Tension-free repair with mesh was applied in six cases (54.5%), with tissue repair in four cases (36.4%). Hernia recurrence was not reported.

**Fig. 4:**
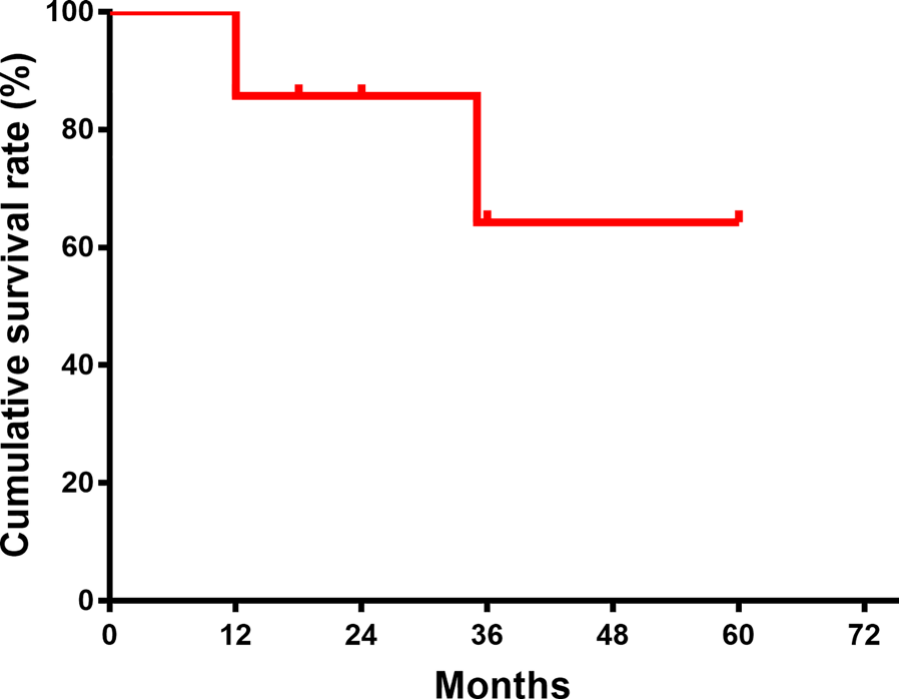
The long-term oncological outcomes of patients with GIST-associated groin hernia. Kaplan–Meier surviving analysis with available follow-up data showed that estimated 5-year overall survival rate was 64.3%

According to included reports, the estimated incidence of GIST-involved groin hernia is less than 0.1%. Follow-up data were received from seven case reports (Table 2). The follow-up period ranged from 18 to 60 months, and two dead cases were recorded during the period. Tumor recurrence in groin was not observed. The Kaplan–Meier surviving analysis suggested an approximate 64.3% overall survival at five years after the incidental diagnosis (Fig. 4).

**Table 2:** Clinical outcomes of included cases

Study	Follow-up period (months)	Hernia recurrence	Inguinal tumor recurrence	Overall survival time (months)
Yuan 2020	18	No	No	18
Ferhatoglu 2018 [12]	18	No	No	12 (Died)
Campbell 2017 [26]	36	No	No	35 (Died)
Agrawala 2017 [27]	–	–	No	–
Massani 2017[10]	36	No	No	36
Tinoco 2015 [28]	–	–	–	–
Zyluk 2015 [16]	60	No	No	Survived
Liu 2014 [29]	24	No	No	Survived
Chen 2013 [30]	60	No	No	Survived
Mulla 2007 [25]	–	–	–	–
Goyal 2003 [31]	–	–	–	–

Data were retrieved or requested from included case reports. “–“ indicates not available data due to missing follow up or no response to data request

GISTs, accounting for 1.0–3.0% of all GI tumors [8], could arise in everywhere of the GI tract. Less common primary sites include the duodenum (4–5%) and rectum (4%). The rare sites include the esophagus (< 5%), Meckel’s diverticulum (< 1%), appendix (< 1%), umbilicus and pituitary (< 1%), with limited case reports available [9–12]. To the best of our knowledge, ten reported cases of a GIST presenting as groin hernia were available (Table 1). The first case of tumor-associated inguinal hernia was reported in 1749 [13]. Of note, the most common inguinal tumors are of colon cancer [14].

In clinical practice, GI hemorrhage (48.9%) and small bowel obstruction (28.3%) were the most frequent presenting symptoms of GISTs [15]. In our case, the patient admitted with acute abdomen and GI bleeding, which was an emergency presentation of GISTs. Unfortunately, his concealed inguinal hernia stemming from tumor seeding due to ruptured GIST was not diagnosed or measured in the first place. It reminds us never to lose sight of groin areas when performing abdominal examinations. One of classical feature is the mass in the sac would be likely diagnosed as inflamed Meckel’s diverticulum [16].

GISTs possess malignant potential and are highly invasive and tend to metastasize to remote organs [17]. Metastasis from primary GIST is frequently recorded, and patients may present with metastatic deposit as the first signs [18]. The most common sites of metastasis are the liver and peritoneum; however, extra-abdominal metastasis is extremely rare. Those uncommon locations include central nervous system, lung, bone, subcutaneous tissues and genitourinary system [18, 19]. Interestingly, lymph node metastasis was rarely occurred.

CT scans played invaluable role in discovering GISTs. Specifically, a modified CT by insufflating CO_2_ to allow a maximum lumen distension of GI wall is strongly recommended for detecting rare site GISTs (esophagus, appendix, rectum, etc.). Additionally, EUS could provide a precise evaluation of submucosal esophageal and gastric tumors. Its main advantage lies on the assessment of origination and invasion of primary tumor, whether the tumor develops from the layers of GI tract or corresponds to an extrinsic compression [20].

Moreover, immunostains with CD117, DOG-1, SDHB and other molecular markers are essential for diagnosis of GIST and facilitate optimization of management of GIST [21]. The main prognostic factors of GISTs are mitotic rate, tumor size and tumor site. These criteria have been incorporated into the risk classification proposed by the Armed Force Institute of Pathology, which gains great popularity in clinical practice [22]. Besides, tumor rupture in abdomen, whether spontaneous or following iatrogenic injury, proved to harbor a risk of recurrence of nearly 100% [23]. Recently, prognostic contour maps are developed, which incorporate the mitotic index and tumor size as continuous non-linear variables, while tumor rupture is considered in addition to tumor site [24].

All irreducible hernias should be thoroughly examined, including physical examination and imaging scans, and then be referred for a surgical assessment. Those hernias may conceal an underlying case that requires urgent investigation and treatment [25]. Suspecting the presence of an incarcerated groin hernia, urgent exploration of inguinal canal is required, followed by an initial evaluation of herniated content. A consideration of laparotomy conversion when the mass is failed to excise through the inguinal incision. To achieve a negative resection margin, at least 2 cm from the gross tumor is required during surgery. However, a palliative resection is not suggested due to its high recurrence rate. As mentioned above, lymph nodes are rarely involved, hence, lymphadenectomy is not necessary during the surgery. As for groin area repair, tension-free herniorrhaphy with non-absorbable mesh is recommended.

## Conclusions

In summary, primary or metastatic GIST could present with symptoms mimicking as a groin hernia. When diagnosing those unusual cases, physicians or surgeons should consider whether malignancy is present, particularly among patients with a history of GIST. Abdominal and pelvic CT scans with enhanced contrast are effective tools for differential diagnoses and subsequent treatments. Never forget to check both groin areas when doing physical examination. Radical tumor resection plus tissue-free hernia repair is an optimal operation for an emergency case. An adjuvant medication targeted to the mutated KIT gene should be strictly followed for high-risk cases.

## Supplementary Information

**Additional file 1: Fig. S1** The primary jejunal GIST and its pathological presentation. (A) primary tumor and intra-abdominal tumor seeding were found; (B) The primary tumor was excised with a safe margin; (C) The tumor-attached intestinal lumen was uninvolved; (D) The gross appearance from middle-incision cut surface showed hemorrhagic necrosis and firm spindle mass with a pseudo-capsule; (E) The hematoxylin–eosin (HE) staining recorded its spindle cell nature.

## Data Availability

The datasets used during the current study are available from the corresponding author on reasonable request.

## Abbreviations

GIST: Gastrointestinal stromal tumor
GI: Gastrointestinal
EUS: Endoscopic ultrasound
CT: Computed tomography
HPF: High power field
IHC: Immunohistochemical
