# Supplementary figures and images for: A concealed inguinal presentation of a gastrointestinal stromal tumor (GIST): a case report and literature review
Source: BMC Surg. 2021 Mar 3;21:111. doi: 10.1186/s12893-021-01088-4 (PMC7931599; doi:10.1186/s12893-021-01088-4)

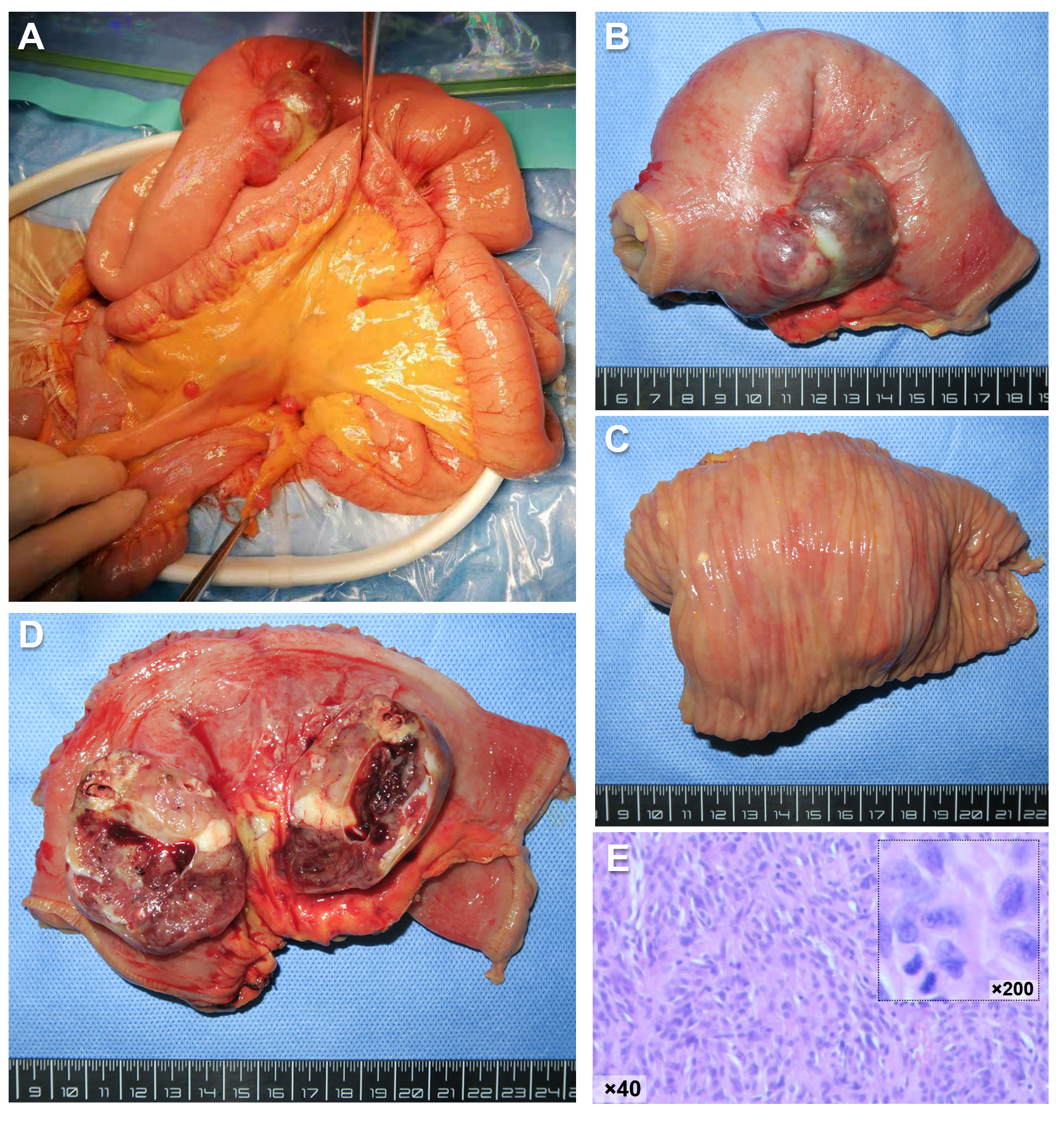

Supplement: Supplementary file 1 — Additional file 1: Fig. S1 The primary jejunal GIST and its pathological presentation. (A) primary tumor and intra-abdominal tumor seeding were found; (B) The primary tumor was excised with a safe margin; (C) The tumor-attached intestinal lumen was uninvolved; (D) The gross appearance from middle-incision cut surface showed hemorrhagic necrosis and firm spindle mass with a pseudo-capsule; (E) The hematoxylin–eosin (HE) staining recorded its spindle cell nature. [file 12893_2021_1088_MOESM1_ESM.tif]
